# Intradural squamous cell carcinoma in the sacrum

**DOI:** 10.1186/1477-7819-7-16

**Published:** 2009-02-11

**Authors:** Tatsuki Fukushima, Yuichi Kasai, Ko Kato, Kozo Fujisawa, Atsumasa Uchida

**Affiliations:** 1 Department of Orthopaedic Surgery, Suzuka Kaisei General Hospital, Tsu City, Mie, Japan; 2 Department of Orthopaedic Surgery, Mie University Graduate School of Medicine, Tsu City, Mie, Japan

## Abstract

**Background:**

Leptomeningeal carcinomatosis occurs in patients with cancer at the rate of approximately 5%; it develops particularly in patients with breast cancer, lung cancer, melanoma, leukemia, or malignant lymphoma. We describe a rare case of leptomeningeal carcinomatosis in which spinal intradural squamous cell carcinoma with no lesions in the cerebral meninges and leptomeninx, was the primary lesion.

**Methods:**

A 64-year-old man complained of sacral pain. Although the patient was treated with analgesics, epidural block and nerve root block, sacral pain persisted. Since acute urinary retention occurred, he was operated on. The patient was diagnosed as having an intradural squamous cell carcinoma of unknown origin.

**Results:**

Since the patient presented with a slightly decreased level of consciousness 2 months after surgery, he was subjected to MRI scanning of the brain and spinal cord, which revealed disseminated lesions in the medulla oblongata. The patient died of pneumonia and sepsis caused by methicillin-resistant *Staphylococcus aureus *5 months after surgery.

**Conclusion:**

We report the first case of a patient with intradural squamous cell carcinoma with unknown origin that developed independently in the sacrum.

## Background

Leptomeningeal carcinomatosis occurs in patients with cancer at the rate of approximately 5%; it develops particularly in patients with breast cancer, lung cancer, melanoma, leukemia, or malignant lymphoma [1-3]. Leptomeningeal carcinomatosis, such as spinal intradural squamous cell carcinoma with no lesions in the cerebral meninges and leptomeninx, occurs very rarely as an independent lesion [4,5]. We present a case of intradural squamous cell carcinoma of unknown origin that developed independently in the sacrum, and a review of published cases.

## Case presentation

A 64-year-old man presented with a chief complaint of sacral pain. His family history was unremarkable. Sacral pain had occurred without the participation of any inducible event 3 months before consultation and had aggravated, resulting in walking difficulty; thus, the patient was admitted for a detailed evaluation. Although the straight leg raising (SLR) test caused no pain, bilateral SLR test until approximately 70° caused sacral pain. Sensation and muscular strength of bilateral lower legs, patellar tendon reflex and achilles tendon reflex were normal and negative results were obtained for Babinski's sign. Although abnormal skin findings such as redness, swelling, and dimple formation around the sacrum were absent, tenderness was identified in the middle of the sacrum. Neither vesicorectal disturbance nor abnormal sensation was apparent in the perineal region, and strength of the anal sphincter, anal reflex and bulbocavernosus reflex were normal. Peripheral blood testing and blood biochemistry showed normal results and the C-reactive protein test was negative. Spinal fluid showed normal cell counts (1 cell/μL) and protein and sugar levels, with no atypical or abnormal cells.

**Figure 1:**
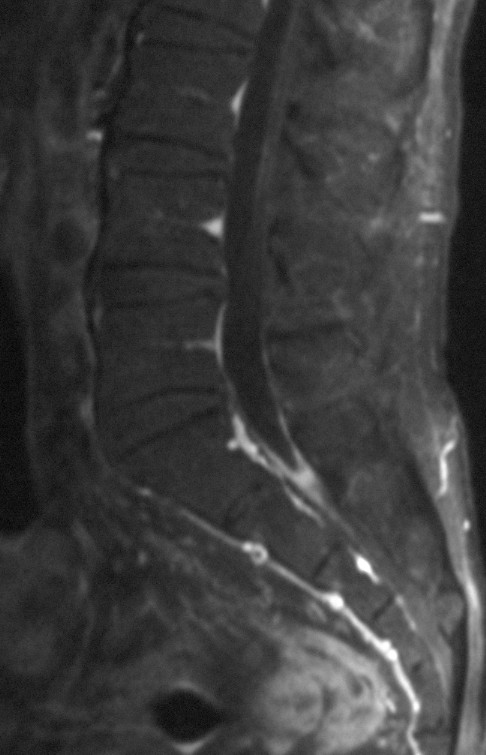
**Sagittal section of Gadolinium-enhanced T1-weighted MRI revealed a V-shaped caudal dural sac of the sacral spine along the sacral dura mater**.

**Figure 2:**
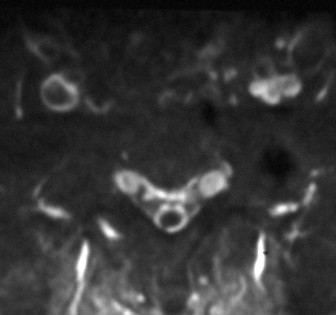
**Axial section of Gadolinium-enhanced T1-weighted MRI demonstrated an annular sac from S1 level to the most caudal region of the dural sac**.

**Figure 3:**
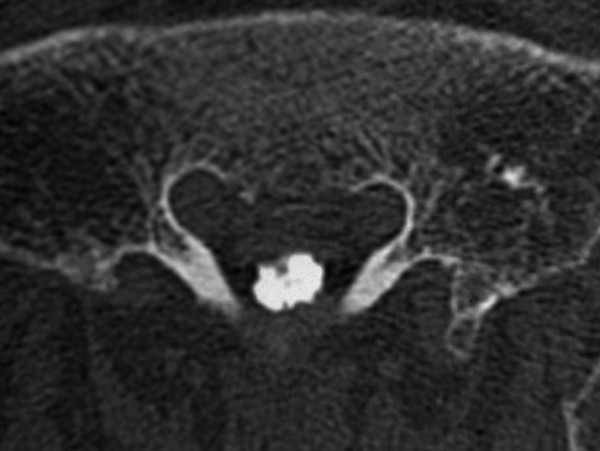
**Myelo-computed tomography showed irregular images in the dural sac wall from S1 level to the most caudal region of the dural sac**.

Plain radiography showed normal images of the lumbosacral spine. Although the magnetic resonance image (MRI) of the lumbosacral spine appeared normal on T1- and T2-weighted images, the sagittal section (Fig. 1) revealed a V-shaped caudal dural sac of the sacral spine along the sacral dura mater; the axial section (Fig. 2) demonstrated an annular sac from the S1 level to the most caudal region of the dural sac on gadolinium-enhanced T1-weighted imaging. Bone scintigraphy showed no abnormalities, with no radio-accumulation in the sacrum. Although myelography revealed no significant abnormalities, myelo-computed tomography (CT) showed irregular images in the dural sac wall from S1 level to the most caudal region of the dural sac (Fig. 3).

**Figure 4:**
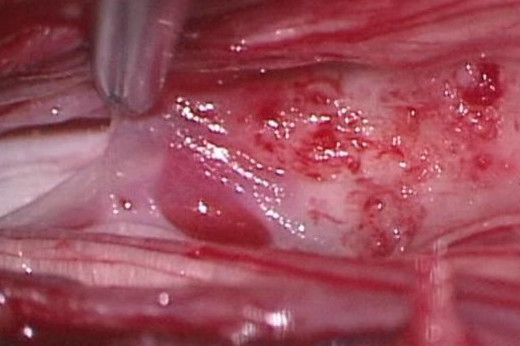
**Operative findings**. White translucent membranous tissues were seen all around the inner wall of the dura mater, firmly attaching to the cauda equine.

Although the patient was treated with analgesics, epidural block and a nerve root block, sacral pain persisted. Since acute urinary retention occurred, he was operated on emergency. The patient underwent a posterior median incision under general anesthesia for a laminectomy of L5, S1, and S2, which revealed neither macroscopic abnormalities of the dura mater nor stenosis of the dural tube. Then the dura was incised from the S1 to S3 level, and white translucent membranous tissues were seen all around the inner wall of the dura mater, firmly attaching to the cauda equina (Fig. 4). The white translucent tissues were carefully detached from the cauda equina and removed to the fullest possible extent. The dura mater was then sutured, and fatty tissues and fibrin glue were placed behind the dura mater before completion of surgery.

**Figure 5:**
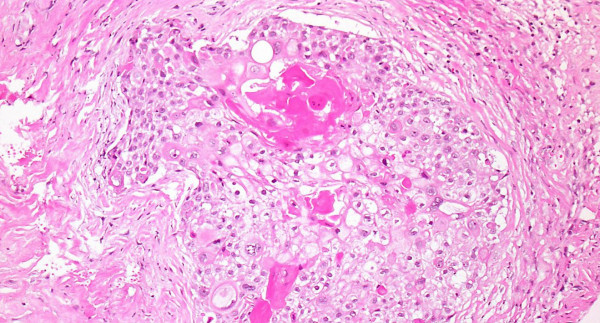
**Histopathological examinations showed thick keratotic lesions with different sizes and mitoses of nuclei, and focal proliferation of atypical squamous cells (Hematoxylin and eosin stain, ×200)**.

The white translucent membranous tissues were diagnosed as squamous cell carcinoma, since histopathological examination showed thick keratotic lesions of different sizes and mitoses of nuclei, and focal proliferation of atypical squamous cells (Fig 5).

**Figure 6:**
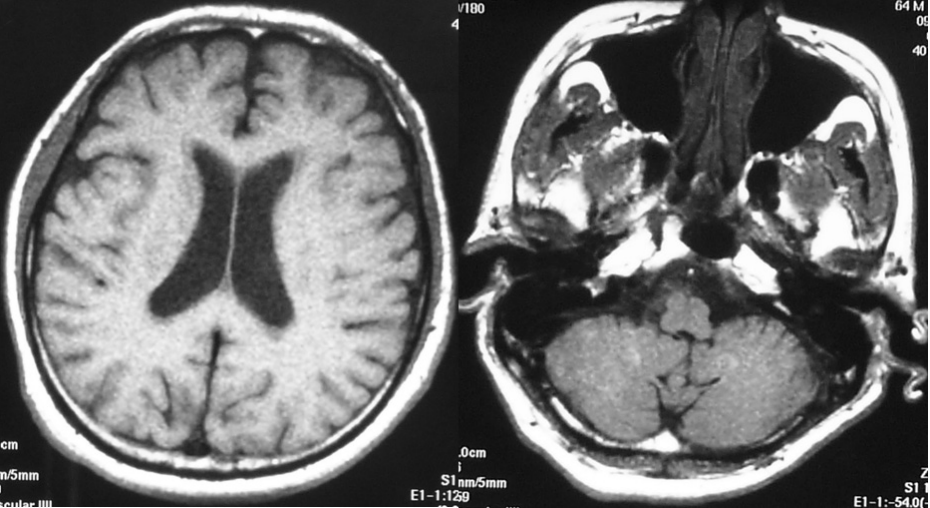
**MRI of the brain showed no abnormal findings immediately after surgery**.

Postoperatively, sacral pain was slightly improved with little improvement of the vesicorectal disturbance. Histopathological results indicated metastatic squamous cell carcinoma of unknown origin and the sacrum was irradiated with 40 Gy. For examination of the whole body, MRI of the brain (Fig 6) and spinal cord, gallium scintigraphy, CT of the chest and abdomen, and positron emission tomography-CT were carried out, but they showed normal findings. Tumor markers including carcinoembryonic antigen, alpha-fetoprotein, carbohydrate antigen 19-9, and squamous cell carcinoma-related antigen were all normal. In addition, dermatologic and proctoscopic examination, and the microscopic examination of the oral cavity, esophagus, and stomach did not reveal any possible origin of the squamous cell carcinoma in this patient.

**Figure 7:**
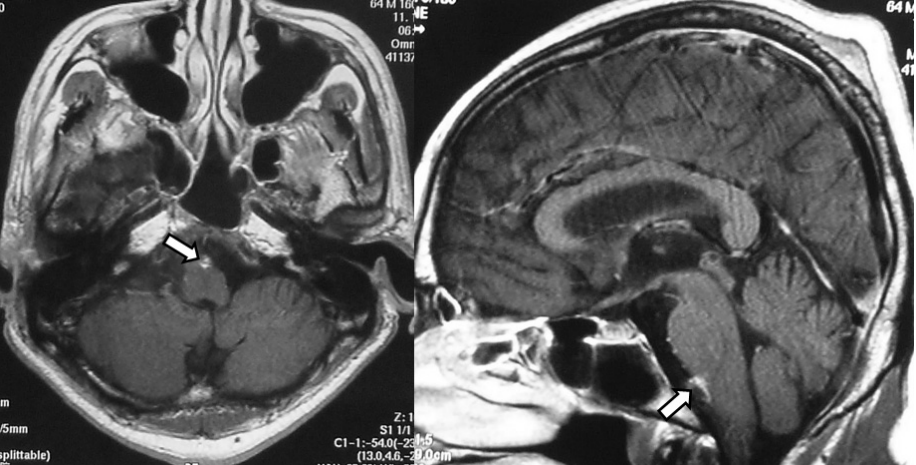
**MRI of the brain was performed again two months after surgery**. This time it revealed disseminated lesions in the medulla oblongata.

Since the patient presented with a slightly decreased level of consciousness 2 months after surgery, MRI of the brain and spinal cord was performed again. This time MRI revealed disseminated lesions in the medulla oblongata (Fig 7). Although radiation with 35 Gy was administered again to the whole brain and lumbar spine, the general condition of the patient gradually deteriorated. The patient died of pneumonia and sepsis caused by methicillin-resistant *Staphylococcus aureus *5 months after surgery.

## Discussion

Intradural squamous cell carcinoma is relatively rare. Furthermore, a computerized search of the Pubmed databases revealed only 8 cases of squamous cell carcinoma with intradural spinal metastases in which the initial symptoms appeared in the spinal cord or cauda equina, but not in the brain (Table 1). Mean age of those patients was 61.1 years (range, 29–77 years; 7 men, 1 woman). Intradural-intramedullar spinal metastasis occurred in 4 of these cases [6-9]. The remaining 4 patients displayed only intradural-extramedullar spinal metastases: a 59-year-old man with an intradural spinal metastasis at L3–L5 [4], a 73-year-old man with intradural spinal metastasis at L1 [10], a 69-year-old man with an intradural-extramedullar thoracic metastasis [11] and a 63-year-old man with intradural spinal metastasis in the lumbar spine [12]. We found no reports of a patient with intradural squamous cell carcinoma in the sacrum. Their primary lesions included the lung in 5 patients, the uterus in 1, the anus in 1, and the supraglottic larynx in 1. Our patient showed dissemination into the spinal fluid in the medulla oblongata two months after surgery and then died without apparent lesions in other organs, not even in the lungs, thus the origin of the squamous cell carcinoma remained unknown. We found the case of a 56-year-old patient with primary (but not metastatic) intradural squamous cell carcinoma on the Pubmed. He had had a spinal dermal cyst in his childhood that transformed into lumbar intradural squamous cell carcinoma later [13]. Although approximately 30 patients who had a cranial epidermal cyst or dermal cyst transformed into squamous cell carcinoma have been reported [14,15], the patient mentioned above was the only one in whom the cyst had transformed into spinal carcinoma. These data indicate that cases of primary intradural squamous cell carcinoma in the spine are very rare. In our patient, there were no apparent primary lesions of squamous cell carcinoma in any organ and no cranial epidermal or dermal cyst was found although cranial MRI revealed metastatic medullary lesions. Thus, we believe that this was a rare case of primary lumbar intradural squamous cell carcinoma in which probably a minute sacral dermal cyst transformed into carcinoma. A definitive diagnosis was, however, impossible to make since the pathological examination performed during sacral surgery revealed neither a cyst nor cyst wall tissues and autopsy was not performed.

**Table 1:** Eight reported cases of squamous cell carcinoma with intradural spinal metastasis in which the initial symptoms appeared in the spinal cord and cauda equina, but not in the brain

Author	Age	Gender	Primary tumor	Involved location of metastasis	
Connolly	62	m	Lung	C4-6	(Intramedullary)

Hirose	57	m	Lung	T6-8	(Intramedullary)

Tashima	77	m	Lung	L1-2	(Intramedullary)

Amin	29	f	Cervix	C4	(Intramedullary)

Cho	59	m	Anal canal	L3-5	(Cauda equina)

Stambough	73	m	Lung	L1	(Cauda equina)

Okamoto	69	m	Lung	Thoracic	(Extramedullary)

Thompson	63	m	Supraglottic larynx	lumbar	(Cauda equina)

Although intradural squamous cell carcinoma is generally treated with chemotherapy, radiation of the brain and whole spinal cord, and tumor removal, these treatments are unsuccessful in most cases. Many patients display early dissemination of tumor cells to the brain and subsequently die from sepsis or factors related to a poor general condition. Similarly, the present patient died approximately 10 months after the onset of symptoms. Among the 9 previously reported cases (8 metastases and 1 primary) with intradural squamous cell carcinoma, the interval between onset and death was given for 6 patients. The mean survival time was 4.2 months (range, 3 weeks to 11 months), indicating the poor prognosis of this disorder.

## Conclusion

We report the first case of a patient with intradural squamous cell carcinoma with unknown origin that developed independently in the sacrum.

## Consent

Written informed consent was obtained from the patient for publication of this case report and accompanying images. A copy of the written consent is available for review by the Editor-in-Chief of this journal.

## Competing interests

The authors declare that they have no competing interests.

## Authors' contributions

TF, KK and KF had a clinical management of this patient. TF drafted the manuscript, did first selection of articles, and assessed the quality of the papers. YK and AU revised the manuscript critically. All authors read and approved the final manuscript.
